# Delays in diagnosis and treatment of pulmonary tuberculosis in Wakiso and Mukono districts, Uganda

**DOI:** 10.1186/1471-2458-14-586

**Published:** 2014-06-11

**Authors:** Esther Buregyeya, Bart Criel, Fred Nuwaha, Robert Colebunders

**Affiliations:** 1 Makerere University School of Public Health, Kampala, Uganda; 2 Institute of Tropical Medicine, Antwerp, Belgium; 3 University of Antwerp, Antwerp, Belgium

**Keywords:** TB treatment delay, Patient delay, Health service delay, TB infection control, Uganda

## Abstract

**Background:**

Delay in tuberculosis (TB) diagnosis may worsen the disease and increase TB transmission. Therefore, timely diagnosis and treatment is critical in TB control. We aimed to assess the treatment delay of pulmonary TB and its determinants in two Ugandan districts where TB infection control (TBIC) guidelines were formerly implemented.

**Methods:**

A facility based cross-sectional study was conducted in Mukono and Wakiso districts. Adult pulmonary TB patients within three months of initiating treatment were included in the study. Delays were categorized into unacceptable patient delay (more than 3 weeks from the onset of cough and the first consultation with a health care provider), health service (more than one week from the first consultation to the initiation of TB treatment) and total delay (more than 4 weeks since the onset of cough). The prevalences as well as predictors for the three delays were determined.

**Results:**

We enrolled 158 sputum positive patients. Unacceptable patient delay was noted in 91 (58%) patients, a health service delay in 140 (88%) patients and a total delay in 140 (90%) patients. An independent predictor for patient delay was male gender (p < 0.001). First visiting a non-public health facility (p = 0.001) was an independent predictor of health service delay.

**Conclusion:**

There is still a significant TB diagnosis and treatment delay in Uganda. Most of the delay was caused by health system delay in the non-public health care sector. There is need for TB advocacy in the community, training of health workers in TBIC and strengthening public-private partnerships in TB control.

## Background

Uganda is among the 22 high tuberculosis (TB) burden countries. It has a treatment success of 67%, default rate of 11% and 50% of TB patients are HIV co-infected [1]. Multi-drug resistant TB (MDR-TB) poses a problem in 1.1% of new cases and 12% of retreatment cases in 2010 [1,2]. One of the contributors to these poor indicators is due to the prolonged delay from the onset of TB disease to the time of diagnosis and initiation of treatment. Delay in diagnosis may lead to progression of disease, increased mortality and TB transmission in the community [3,4]. Therefore, timely diagnosis and treatment is critical in TB control. Studies from Uganda and Tanzania have shown median diagnostic delays varying from 8 to 13 weeks [5-7]). In two systematic reviews, it was found that diagnosis and treatment delays of TB occurs more often in patients with smear negative TB, HIV infection, living in a rural area and or far away from a health unit and in patients consulting with traditional healers [8,9].

In 2009, WHO released TB infection control (TBIC) guidelines [10]. Early detection and prompt effective TB treatment are the hallmark of a successful TBIC. These guidelines recommend minimizing the time spent in health facilities, by ensuring that people with TB symptoms are rapidly identified and if found to have TB, promptly treated. Exposure to people who are infectious should be minimized by reducing the number of outpatient visits [10]. Furthermore, encouraging people to rapidly seek care if they have symptoms suggestive of TB [10]. In 2008, the Uganda Ministry of Health (MOH) in collaboration with the TB Union, through support from the Tuberculosis Control Assistance Programme implemented a two year TBIC project in 12 districts in Uganda including Mukono district. In 2010, the Ugandan MOH implemented TBIC in Wakiso district and in 2011 released national TBIC guidelines, adopted from the WHO [11]. To our knowledge no other study in Uganda has examined TB treatment delays since the implementation of TBIC. Our study assessed the magnitude of patient, health service and total delay; and their determinants in a predominantly rural setting in Uganda.

## Methods

### Design and study setting

A cross-sectional survey among patients with pulmonary TB was conducted in Mukono and Wakiso districts. These two central districts surrounding the capital Kampala are partly semi-urban but predominantly rural [12]. The reported case detection rates of new pulmonary positive TB in Mukono and Wakiso districts were 40.1% and 38.5%, respectively in 2008. Mukono has 25 and Wakiso 26 healthcare facilities currently providing TB services. The study was conducted among patients attending selected health centre IV and hospital level facilities; government and one Private-Not-For Profit (PNFPs) facilities. These facilities were purposely selected because they handle most of the TB patients in their geographical locations. In Uganda, TB services are provided at health centre III (sub-county level) and above. TB services are, at least in principle, free of charge in all government facilities, while in PNFPs, patients pay for consultation and investigations, but after diagnosis the TB drugs are provided for free. Diagnosis of pulmonary TB is by smear microscopy. According to TBIC guidelines, patients presenting to the facility with cough of two or more weeks have to submit two sputum samples in the form of a spot-sample and morning sample the following day. Spot specimens are collected on the day the patient suspected to have TB is seen at the facility, morning samples are collected early in the morning of the second day [13]. A patient is diagnosed with TB if one sputum smear is positive. Treatment should be initiated on the same day after receipt of a positive result, ideally within 24 hours after submission of the first sputum specimen [11]. TB/HIV collaborative activities exist in the study districts and so all HIV patients are supposed to be screened for TB and TB patients counselled and tested for HIV.

### Study participant selection

We aimed to recruit patients 18 years and above, who started treatment in the 3 months preceding the start of the survey. Eligible participants were asked to participate in the study as they visited the TB clinics for drug refill. Patients were consecutively enrolled and interviewed using a structured questionnaire. Patients previously treated for TB and those with extra-pulmonary TB were excluded from the study. Only sputum smear positive patients were included in the study. All of them were out-patients.

An interviewer-administered structured questionnaire was used, adapted from a previous study on TB diagnosis delay conducted in public primary health facilities in Kampala city, Uganda [7]. The questionnaire was translated to Luganda. The following information was collected: demographic and socio-economic characteristics, TB symptoms and duration in number of weeks before seeking care outside home, the date of patient’s first consultation with a health care provider and any other consultations with subsequent providers, if any, before TB treatment was started, distance to the health facility where TB diagnosis was made, and date of starting treatment. In addition patients were asked whether TB is curable and if they had tested for HIV before being diagnosed of TB. Participants were enrolled between December 2012 and March 2013.

### Definitions and measures

Patient delay was defined in a similar way as in previous studies in Uganda, as the time interval in weeks between onset of cough and the first consultation with any health care provider [5-7]. A health care provider was defined as any person consulted by the patient about his/her sickness who prescribed any form of medication (Table 1). These included dispensers, pharmacists, medical staff and herbalists/traditional healers. Unacceptable patient delay was defined as a delay of more than 3 weeks since the onset of cough (i.e. one week after the 2 weeks of cough when one is considered a TB suspect) [14,15]. Health service delay was defined as the time in weeks from the first consultation to the initiation of the TB treatment. Unacceptable health service delay was defined as a delay of more than 1 week. The total delay was the sum of both, patient and health service delay [6,7]. Unacceptable total delay was defined as a delay of more than 4 weeks [5,14]. Cough was chosen to define delay because it is the cardinal symptom of pulmonary TB. The estimated sample size of 158 patients was obtained assuming, from prior local study that 91% would have a total delay of more than 4 weeks [5]. This took into consideration a power of 80%, 95% confidence interval and a 20% adjustment for missing and non response.

**Table 1:** Patient, health service and total delays in published studies and current study

**Reference**	**Definitions used**			**Prevalence of delay**	**Median/mean total delay**
	**Patient delay**	**Health service/facility delay**	**Total delay = Patient + health facility delay**		
Kiwuwa et al. (2005) Uganda [5]	- The time interval between symptom onset and the first medical consultation.	- The time taken from the first medical consultation to when the diagnosis was confirmed and treatment started.	- > 4 weeks was considered as prolonged/ unacceptable total delay.	- Patient delay: not specified	-Median: 12 weeks, IQR not specified
				- Health service delay: 74%	
	- A health provider was defined as any person consulted by the patient about his/her sickness, who prescribed any form of medication. These included dispensers, pharmacists, medical staff and herbalists or traditional healers.			- Total delay: 91%	
		- Health service delay was when there was a delay of > 4 weeks between the initial contact with the health provider and the start of TB treatment.			
	- Interval of > 2 weeks was considered as long patient delay.				
Ngadaya et al. (2009) [6] Tanzania.	- The time interval between the day of experiencing for the first time one of the pulmonary symptoms to the day the patient sought medical advice for the first time.	- A time interval between first consultations at a health facility to the day the treatment was initiated.	- The sum of the patient and health facility delay.	- Health facility: 59%.	- Mean: 125.5 (SD98.5) days
				- Patient and total delay not specified.	
					- Median: 90 days, IQR not specified
			- A time interval > 35 days.		
	- Interval of > 30 days was considered as patient delay.	- A time interval of > 5 days was considered as health facility delay			
Basnet et al. (2009) Nepal [16]	- The time interval from the appearance of the first symptoms of tuberculosis until the first visit to any formal health care facility (health centres, hospitals or DOTS centres).	- The time interval from the first consultation at any formal health facility until the date of diagnosis.		Not specified.	- Median: 60 days, IQR not specified
Sendagire et al. (2010) [7]	- The time in weeks from the onset of cough to a first consultation with any health care provider.	- The time in weeks from the first consultation to initiation of treatment.	- Prolonged total delay was defined as a delay of > 14 weeks.	- Prolonged patient delay: 19%	-Median: 8 (IQR 4-12) weeks
				- Prolonged health service delay: 29%	
	- Prolonged patient delay was defined as a delay of > 8 weeks.				
		- Prolonged heath facility was defined as a delay of > 6 weeks.			
				- Prolonged total delay: 24.1%	
Current study	-The time interval in weeks between onset of cough and the first consultation with any health care provider (dispensers, pharmacists, medical staff and herbalists/traditional healers).	-The time in weeks from the first consultation to the initiation of the TB treatment.	Unacceptable total delay was defined as > 4 weeks.	- Unacceptable patient delay: 58%	- Median total delay of 16 (IQR 9-30) weeks
				Unacceptable health service delay: 89%	
				- Total delay: 91%	
		- Unacceptable health service delay was defined as a delay of more than 1 week.			
	- Unacceptable patient delay was defined as a delay of more than 3 weeks since the onset of cough.				

≠IQR = Interquartile range.

*SD = Standard deviation.

### Quality control

The questionnaire used was pre-tested. Research assistants were trained and supervised during data collection. Duration of symptoms, date of initiation of treatment were counterchecked in the medical records. Data was double entered into a computer, and the two copies of the data verified.

### Ethical considerations

The research protocol was approved by the institutional review board of the Makerere University School of Public Health and the Uganda National Council for Science and Technology (Ref.nr. HS 880). Permission from the in-charges of the health facilities was obtained. Informed written consent was obtained from the participants at the time of data collection. Respondents were assured of confidentiality.

### Data management and analysis

Data were entered into Epi-info software version 6 and exported to Stata software version 10 for analysis. Health service delay (in days) was computed from the date of first seeking care with cough symptom to when treatment was initiated. It was later converted into weeks by diving by 7. Descriptive statistics were computed, such as frequency distributions, median and interquartile ranges (IQRs). The normality of the delays was checked using Shapiro test and were all found not be normally distributed. Bivariate analysis using Chi-square test and prevalence ratios (using generalized linear model with a Poisson link) were performed. Multivariate analysis was used to explore the predictors of the outcome variables (patient, health service and total delay). Unacceptable patient delay, health service delay and total delay were compared for different sub-groups using prevalence ratios and 95% CI. To identify factors independently associated with unacceptable patient, health service and total delays, a multivariate analysis with delay time dichotomized was performed. Possible predictors (age, sex, residence, type of health provider first visited, education level and distance to the facility of diagnosis) were evaluated in bivariate associations with each type of delay. Variables associated with delays in the bivariate analysis (p ≤ 0.2) were included in the model. Statistical significance was taken as p < 0.05. We adjusted for the clustering effect at both bivariate and multivariate analysis.

## Results

**Figure 1:**
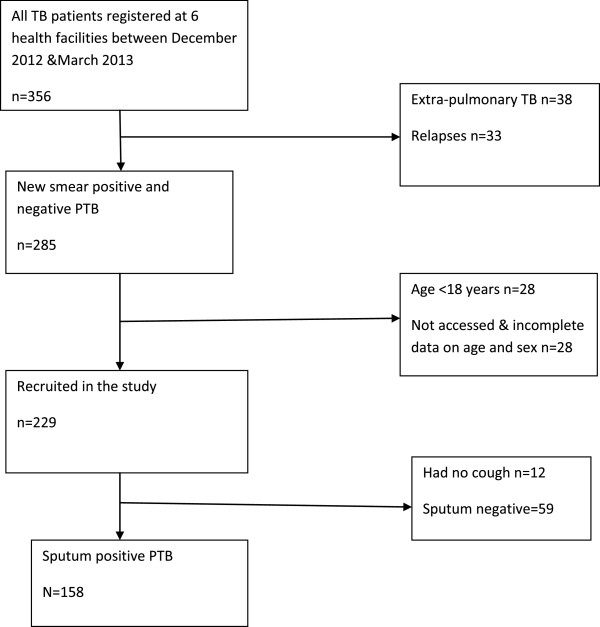
Flowchart showing the patients included in the analysis of treatment delay in Mukono and Wakiso districts, Uganda.

A total of 229 TB patients were prospectively enrolled from 6 health facilities (3 hospitals and 3 health centre IVs). Seventy one patients were excluded from further analysis because they were sputum negative (59) and did not have cough as one of their presenting symptoms (12), leaving 158 patients (Figure 1). The median age was 30 (IQR 25.5-38) years. Ninety six (61%) patients were males, 78 (49%) were married and 13 (8%) had no formal education, (Table 2). Patients’ median family size was 4 (IQR3-7) and they were generally living in one living room. More than half (102; 65%) of the patients lived less than 60 minutes walking distance to the facility where the TB was diagnosed. The majority of the patients, 104/157 (65%) had ever tested for HIV before TB diagnosis (Table 2). One hundred forty three (97%) respondents accepted to share their results, with 31% (45/143) reporting being HIV positive. In addition to cough, at the time of diagnosis, patients had the following symptoms: chest pain 94/157 (60%), fever 85/157 (54%), weight loss 80/157 (51%), loss of appetite 67/156 (43%) and haemoptysis 28/158 (18%).

**Table 2:** Socio-demographic and clinical characteristics of the study patients

**Variable**	**Total n (%)**
**Sex (n = 158)**	
Female	62 (39)
Male	96 (61)
**Age years (n = 158)**	
15-24	36 (23)
25-34	65 (41)
35-44	38 (24)
>44	19 (12)
**Monthly salary income in Ugandan shillings (n = 156)**	
≤20,000	39 (25)
> 20,000	117 (75)
**Cost for one way journey to the facility of diagnosis (n = 158)**	
≤2,000	104 (66)
>2,000	54 (34)
**Marital status (n = 158)**	
Married	78 (49)
Single	38 (24)
Separated/widowed	42 (27)
**Residence (n = 158)**	
Rural	57 (36)
Semi-urban	101 (64)
**Education level (n = 158)**	
No formal education	13 (8)
Primary	73 (46)
Secondary and above	72 (46)
**Patient knows TB can be cured (n = 158)**	
Yes	82 (52)
No	76 (48)
**Tested for HIV before TB diagnosis (n = 158)**	
Yes	104 (66)
No	54 (34)

### Health care seeking behaviour

Only 35/158 (22%) patients first sought care from a public health facility. Patients who first sought care from non-public health facilities were similar to those who first visited a public health facility regarding sex, marital status, residence and having been tested for HIV before TB diagnosis. Patients of different age category and education level tended to seek care differently. Non-public health facilities were visited by 123 (78%) of the 158 patients: private clinics by 65 patients (41%), drug shops by 17 patients (11%), a private pharmacy by 16 patients (10%), traditional healers/herbalists by 15 patients (9%) and PNFPs by 10 patients (6%). Reasons for not seeking care from public health facilities were available for 119 patients. These included: long waiting hours in 33/119 (28%), the facility being far away in 35/119 (29%), a bad previous experience in 20/119 (17%) and thinking that their illness was not that serious 31/119 (26%). Majority of the respondents (143/158 (91%)), made the first consultation because of cough. The services provided by the health care provider during this first visit were: offering non-TB medical treatment in 128/156 (82%) patients, sputum examination in 24/156 (15%) patients, x-ray in 2/156 (1%) patients, and referring the remaining patients.

### Delays and determinants

#### Patient delay

The median patient delay was 4 (IQR 2-8) weeks. In 91 respondents (58%) there was unacceptable patient delay. The majority of the patients (81/91) who delayed seeking care, thought that their symptoms would spontaneously go away, while 11/91 (9%) did not perceive a time span of more than 3 weeks as problematic. The remaining patients delayed to seek care due to economic constraints and lack of family support. In our bivariate analysis, patient delay was significantly associated with being male (p < 0.001), but not with age, education level, salary, distance to the facility of diagnosis, residence, marital status or having tested for HIV before the current TB diagnosis (Table 3). In our multivariate analysis, after controlling for age, sex, education level, and marital status, male patients were more likely to delay seeking care (adjusted Prevalence ratio [aPR] 1.61(1.43-1.82).

**Table 3:** Predictors of unacceptable patient delay (>3 weeks)

**Variable**	**Unacceptable patient delay**		**Univariate analysis**	**Multivariate analysis**	**P**
	**Yes**	**No**	**Crude PR 95% CI**	**Adjusted PR 95% CI**	
**Sex**					
Female	26/62 (42)	36/62 (58)	1	1	
Male	65/94 (69)	29/94 (31)	1.64 (1.41-1.91)	1.61 (1.43-1.82)	<0.001*
**Age group**					
15-34	52/100 (52)	48/100 (48)	1	1	
35 & above	39/56 (70)	17/56 (30)	1.33 (0.98-1.82)	1.00 (0.85-1.18)	0.97
**Education level**					
No formal education	9/13 (69)	4/13 (31)	1	1	
Primary	49/72 (68)	23/72 (32)	0.98 (0.63-1.52)	0.92 (0.66-1.27)	0.61
Secondary & above	33/71 (46)	38/71 (54)	0.67 (0.42-1.06)	0.70 (0.45-1.09)	0.12
**Income in Ugandan shillings**					
0-20,000	21/38 (55)	17/38 (45)	1	-	-
>20,000	69/116 (59)	47/116 (59)	1.07 (0.85-1.34)	-	-
**Distance to facility of diagnosis**					
Within 5km	48/85 (56)	37/85 (44)	1	-	-
Over 5Km	43/71 (61)	28/71 (39)	1.07 (0.83-1.38)	-	-
**Type of facility first visited**					
Non-Public(private)	69/122 (57)	53/122 (43)	1	-	_
Public	22/34 (65)	12/34 (35)	1.14 (0.83-1.57)		
**Patient knows TB can be cured**					
No	48/81 (59)	33/81 (41)	1	-	-
Yes	43/75 (57)	32/75 (43)	0.96 (0.86-1.08)		
**Tested for HIV before**					
Yes	59/102 (58)	43/102 (42)	1	_	_
No	32/54 (59)	22/54 (41)	1.02 (0.81-1.29)		
**Residence**					
Rural	35/57 (61)	22/57 (39)	1	_	_
Semi-urban	56/99 (57)	43/99 (43)	0.92 (0.68-1.23)		

*Statistically significant.

#### Health service delay

The median reported health system delay was 11 (5-24) weeks. In the majority of the patients 140/158 (89%) there was an unacceptable health service delay. The median number of visits to all sorts of health care providers before being diagnosed was 3 (IQR3-9). Almost half of the patients who delayed 60/140 (43%), lived beyond 5 km from the facility where diagnosis was made (Table 4). In our bivariate analysis, health service delay was significantly associated with first seeking care in a non-public health facility (p < 0.001). In multivariate analysis, after controlling for age, sex, distance to the facility of diagnosis, education level and type of facility visited first, patients who first sought care in the public health facility were significantly less likely to have unacceptable health service delay, (aPR 0.62; 0.47-0.83).

**Table 4:** Predictors of unacceptable health service delay (>1 week)

**Variable**	**Unacceptable health service delay**		**Univariate analysis**	**Multivariate analysis**	**P**
	**Yes**	**No**	**Crude PR 95% CI**	**Adjusted PR 95% CI**	
**Sex**					
Female	57/62 (92)	5/62 (8)	1	1	
Male	83/96 (86)	13/96 (14)	0.94 (0.85-1.03)	0.98 (0.91-1.05)	0.63
**Age group**					
<=34	92/101 (91)	9/101 (9)	1	1	
>=35	48/57 (84)	9/57 (16)	0.92 (0.84-1.00)	1.02 (0.96-1.09)	0.44
**Monthly income in Ugandan shillings**					
≤20,000	35/39 (90)	4/39 (10)	1	-	-
>20,000	104/117 (89)	13/117 (11)	1.01 (0.93-1.09)		
**Education level**					
No formal education	10/13 (77)	3/13 (23)	1	1	
Primary	63/73 (86)	10/73 (14)	1.12 (0.82-1.51)	0.99 (0.77-1.28)	0.99
Secondary and above	67/72 (93)	5/72 (7)	1.12 (0.92-1.57)	1.02 (0.80-1.30)	0.83
**Marital status**					
Married	68/78 (87)	10/78 (13)	1	-	-
Single	34/38 (89)	4/38 (11)	1.02 (0.90-1.16)		
Separated/Widow/Divorce	38/42 (90)	4/42 (10)	1.03 (0.94-1.13)		
**Distance to facility of diagnosis**					
Within 5km	80/86 (93)	6/86 (7)	1	1	
Over 5Km	60/72 (83)	12/72 (17)	0.89 (0.788-1.01)	0.93 (0.82-1.06)	0.29
**Type of facility first visited**					
Non-Public (private)	119/123 (97)	4/123 (3)	1	1	0.001*
Public	21/35 (60)	14/35 (40)	0.62 (0.47-0.81)	0.62 (0.47-0.83)	
**Tested for HIV before**					
Yes	91/104 (88)	13/104 (13)	1	-	-
No	49/54 (91)	5/54 (9)	1.03 (0.94-1.13)		
**Residence**					
Rural	50/57 (88)	7/57 (12)	1	_	_
Semi-urban	90/101 (89)	11/101 (11)	1.01 (0.89-1.15)		

*Statistically significant.

#### Total delay

Data for total delay was available for only 154 patients. The median total delay from onset of cough until start of anti-TB treatment was 16 (9-30) weeks. In the majority of the patients 140/154 (91%) there was an unacceptable total delay. In a chi-square test unacceptable total delay was significantly associated with first visiting a non-public health facility (p = 0.05) (Table 5). Health service delay was the greatest contributor (75%) to total delay. In our multivariate analysis being male and first seeking care from non-public health facility were more likely to have unacceptable total delay, though not significant (Table 5).

**Table 5:** Predictors of unacceptable total delay (>4 weeks)

**Variable**	**Unacceptable Total delay**		**Univariate analysis**	**Multivariate analysis**	
	**Yes**	**No**	**Crude PR 95% CI**	**Adjusted PR 95% CI**	**P**
**Sex**					
Female	53/61 (87)	8/61 (13)	1	1	
Male	87/93 (94)	6/93 (6)	1.07 (0.96-1.20)	1.09 (0.99-1.20)	0.073
**Age group**					
≤ 34	89/99 (90)	10/99 (10)	1	_	_
≥35	51/55 (93)	4/55 (7)	1.03 (0.94-1.12)		
**Salary income in Ugandan shillings**					
≤20,000	35/37 (95)	2/37 (5)	1	_	_
>20,000	104/115 (90)	11/115 (10)	0.95 (0.88-1.03)		
**Marital status**					
Married	68/75 (91)	7/75 (9)	1	_	_
Single	34/38 (89)	4/38 (11)	0.98 (0.92-1.05)		
Separated/Widowed	38/41 (93)	3/41 (7)	1.02 (0.88-1.17)		
**Education level**					
No formal education	12/13 (92)	1/13 (8)	1	_	_
Primary	66/71 (93)	5/71 (7)	1.00 (0.90-1.12)		
Secondary and above	62/70 (89)	8/70 (11)	0.95 (0.78-1.17)		
**Distance to facility of diagnosis**					
Within 5km	77/85 (91)	8/85 (9)	1	_	_
Over 5Km	63/69 (91)	6/69 (9)	1.00 (0.89-1.12)		
**Type of facility first visited**					
Non-Public (private)	112/120 (93)	8/120 (7)	1	1	_
Public	28/34 (82)	6/34 (18)	0.88 (0.74-1.05)	0.86 (0.74-1.01)	0.078
**Tested for HIV before**					
Yes	91/104 (88)	13/104 (12)	1	-	-
No	46/54 (91)	5/54 (9)	1.02 (0.91-1.15)		
**Residence**					
Rural	50/55 (91)	5/55 (9)	1	_	_
Semi-urban	90/99 (91)	9/99 (9)	1.00 (0.88-1.13)		

## Discussion

This study performed in two Ugandan districts highlights the unacceptable delay from onset of cough to initiation of TB treatment. More than half of the patients delayed to seek medical care in the presence of cough for more than 3 weeks. Health service delay was the biggest contributor to the total delay. Patient delay was significantly associated with being male. Health service delay was significantly associated with first seeking care from a non-public health facility.

Three quarters of the total delay was due to health service delay. This proportion of the total delay is similar to what was found in a study among patients at the national referral hospital in Kampala [5]. Sendagire et al., on the other hand, in a study in Kampala City Councils clinics observed that patient and health service delay equally contributed to the delay [7]. The long health service delay in our study shows that there are many missed chances for TB diagnosis. While according to the Ugandan National TBIC guidelines, a patient with cough for 2 weeks or more should be investigated for TB [11], in our study only 15% of such patients had a sputum examination done on their first visit to the health care provider. The unacceptable health service delay is an indication of a weak performing health system. This may be because of poor knowledge of health workers about TB, resulting into a low index of suspicion for TB, and/or lack of staff, infrastructure to diagnose and treat TB. This long health service delay means that a significant number of patients remain infectious. This puts other patients in the health facilities and health care workers (particularly HIV infected) at risk of getting nosocomial TB, in addition to transmitting TB to the household members. The finding of unacceptable health service delay being associated with first seeking care from non-public health facilities (non-TB health care providers) is similar to what has been reported in other studies [9,17]. Only public health facilities and a few private providers provide TB treatment in Uganda [7]. Therefore, non-public health facilities like pharmacies, drug shops, some private clinics and traditional healers/herbalist which do not regularly treat TB patients, may have a lower index of suspicion for TB or even no knowledge at all, compared to public facilities. Training of health workers on new guidelines like the TBIC guidelines, are mainly organized in public facilities. Thus seeking care first with a private care provider results in delays in diagnosis and treatment of TB due the longer path from the initial visit to finally accessing a TB service provider (which is usually a public health facility) [17].

The median total delay of 16 weeks observed in our study is close to what was found in other studies from Pakistan and Tanzania [18,19] though lower than what was reported in Kampala City Councils clinics in Uganda [7]. The longer total delay observed in our study compared to previous studies conducted in Uganda [5,7] can be explained by better access to health facilities in the urban setting. Comparing treatment delay between different studies is difficult because they often used different definitions (Table 1). According to the WHO guidelines and the Ugandan MOH TBIC guidelines, patients with cough for 2 weeks and above should be investigated for TB within 24 hours [10,11]. Therefore, in our study we considered a delay of 3 weeks as unacceptable patient delay and a delay of more than one week as unacceptable health service delay. Adhering to these shorter delays will result in low TB transmission and thus improved TB control.

Almost half of the patients sought care within the first three weeks of cough. This is lower than what was observed in a national Ugandan referral hospital [5], where 70% of patients contacted a health care provider within two weeks of cough. This could also be explained by better access to health services in an urban setting, compared to the rural setting where our study was conducted. Almost 10% of our patients first sought care from traditional healers/herbalists. This is consistent with the findings in South Africa [20]. This highlights the need to involve traditional healers in TB control, since a substantial proportion of patients seek care from them. The patient delay observed in this study is comparable to those reported by other studies in sub-Saharan Africa [5,7,21,22]. Patient delay in this study contributed least towards total delay. This is contrary to what was observed in a study from Tanzania where patient delay contributed to 90% of the total delay [23]. However the Tanzanian study used the following definition of patient delay “the period between onset of symptoms and reporting to a health facility.” In our study we defined patient delay as the period between onset of symptoms and seeking care from any health care provider. We used “any health care provider” because of the WHO recommendation to involve all health care providers in TB control [2]. Patient delay was significantly associated with being male, unlike what was observed by Mfinanga in Tanzania [24]. On the other hand, Johansson et al. in Vietnam reported that men were more likely to neglect symptoms and to seek care only when the disease gets into an advanced stage [25]. This has also been reported in HIV care, where men have been found to present with advanced disease and were more likely to be lost to follow-up [26,27].

Unacceptable health service and total delay were associated with visiting non-public health facilities. To reduce these delays, coordination between the National TB and Leprosy Programme (NTLP) and the private sector is critical for TB control [2,28]. Efforts to engage with private providers have already started in Uganda and this resulted in improved case detection in an urban setting [29]. But pharmacies, drug shops and traditional healers need to be brought on board. This can be achieved by the use of incentives to encourage private care providers to refer all TB suspects to a diagnostic facility [2]. However, there is a need to explore more sustainable ways on how to involve them, more particularly the traditional healers. A public-private mix in TB control is in line with the Stop TB Strategy [2]. Furthermore, there is also a need to train all health care providers to raise their level of suspicion for TB. In addition, community TB advocacy and sensitization, which is advocated in TBIC is necessary to improve TB awareness among the people and thus result in early care seeking for TB symptoms. Rolling out rapid diagnostics would also help in reducing diagnostic delay. This could for instance be done by use of the gene X-pert [2,30,31], but also by taking two spot sputum specimens for smear microscopy on the same day rather than on different days [32], so as to reduce patients failing to return in time or even being lost to follow up.

Our study has limitations. Being a retrospective study, imprecise estimates of delay due to recall bias among the patients could have influenced our results. This was minimised by the use of TB treatment cards and case notes. In addition, we asked patients if they thought TB was curable, before they started on treatment. This could have introduced a bias considering the health education patients are given before they start and also in the course of the treatment. We may have also introduced a selection bias by only including outpatients. The study was carried out in a few facilities (health centre IVs and hospitals) and mainly government, so this limits the generalizability of our findings. The picture could be different in lower level facilities and in private facilities. Last but not least, length of cough was used as an indicator for patient delay. However, it can start in an insidious manner and so the timing of onset of cough can vary among respondents depending on the manner they gauge the seriousness of the symptom. Moreover, we did not asses treatment delay of patients with smear negative pulmonary TB and patients with extrapulmonary TB.

## Conclusions

This study reveals that there is still a significant TB treatment delay problem in Uganda. Our study underscores the need to involve all health care providers in TB control. TB treatment delay can be seen as a proxy indicator of health system performance. Our results point to the fact that the public sector seems to be doing better than the various private providers that operate in rural Uganda. The range of relevant actions to be considered could include: health education on TB and TBIC, further decentralization of health services, appropriate training of health care providers in TBIC and public-private partnership in TB control. Research is needed on why there is prolonged health service delay and how private care providers including traditional healers can be effectively engaged by national TB control programmes.

## Competing interests

The authors declare that they have no competing interests.

## Authors’ contributions

EB, BC, FN, RC were involved in the development of the proposal. EB carried out field work. EB, FN, and RC undertook data analysis. EB, FN, RC, and BC drafted the manuscript and approved the final draft.

## Pre-publication history

The pre-publication history for this paper can be accessed here:

http://www.biomedcentral.com/1471-2458/14/586/prepub

## References

[B1] WHO Global tuberculosis control 2011 Geneva: World Health Organization http://www.who.int/tb/publications/global_report/2011/gtbr11_full.pdf

[B2] WHO Global tuberculosis control 2012 Geneva: World Health Organization http://www.who.int/tb/publications/global_report/gtbr12_main.pdf

[B3] Uys PW Warren RM van Helden PD A threshold value for the time delay to TB diagnosis PLoS One 2007 2 8 e757 10.1371/journal.pone.0000757 17712405 PMC1942086

[B4] WHO Tuberculosis programme fact sheet 1996 Geneva: World Health Organization http://www.infocenter.nercha.org.sz/sites/default/files/infocenter_db/ELDOCS/TBHandbook.pdf

[B5] Kiwuwa SK Karamagi C Mayanja KH Patient and health service delay in pulmonary tuberculosis patients attending a referral hospital: a cross-sectional study BioMedCentral Public Health 2005 5 122 1 7 10.1186/1471-2458-5-122 PMC1310609 16307685

[B6] Ngadaya ES Mfinanga GS Wandwalo ER Morkve O Delay in tuberculosis case detection in Pwani region. Tanzania. A cross sectional study BMC Health Serv Res 2009 9 196 10.1186/1472-6963-9-196 19863823 PMC2774679

[B7] Sendagire I Schim Van Der Loeff M Mubiru M Konde-Lule J Cobelens F Long delays and missed opportunities in diagnosing smear-positive pulmonary tuberculosis in Kampala, Uganda: a cross-sectional study PLoS One 2010 5 12 e14459 10.1371/journal.pone.0014459 21206746 PMC3012078

[B8] Finnie RK Khoza LB van den Borne B Mabunda T Abotchie P Mullen PD Factors associated with patient and health care system delay in diagnosis and treatment for TB in sub-Saharan African countries with high burdens of TB and HIV Trop Med Int Health 2011 16 4 394 411 10.1111/j.1365-3156.2010.02718.x 21320240

[B9] Storla DG Yimer S Bjune GA A systematic review of delay in the diagnosis and treatment of tuberculosis BMC Public Health 2008 8 15 10.1186/1471-2458-8-15 18194573 PMC2265684

[B10] WHO WHO policy on TB infection control in health-care facilities, congregate settings and households 2009 Geneva: World Health Organization http://whqlibdoc.who.int/publications/2009/9789241598323_eng.pdf 24432438

[B11] MOH Uganda national guidelines for tuberculosis infection control in health care facilities, congregate settings and households 2011 Kampala: Ministry of Health

[B12] UBOS Uganda national household survey 2009/10 Kampala: Uganda Bureau of Statistics

[B13] Ministry of Health Programme NTaL Ministry of health manual of the national tuberculosis and leprosy programme 2010 2 Kampala: Ministry of Health 22 23

[B14] Lambert ML Van der Stuyft P Delays to tuberculosis treatment: shall we continue to blame the victim? Trop Med Int Health 2005 10 10 945 946 10.1111/j.1365-3156.2005.01485.x 16185227

[B15] WHO Treatment of tuberculosis: guidelines for national programmes second edition 1997 Geneva, Switzerland: World Health Organization http://whqlibdoc.who.int/hq/1997/WHO_TB_97.220.pdf

[B16] Basnet R Hinderaker GS Enarson D Malla P Morkve O Delay in the diagnosis of tuberculosis in Nepal BioMedCentral Public Health 2009 9 236 1 5 10.1186/1471-2458-9-236 PMC2716339 19602255

[B17] Van Wyk SS Enarson DA Beyers N Lombard C Hesseling AC Consulting private health care providers aggravates treatment delay in urban South African tuberculosis patients Int J Tuberc Lung Dis 2011 15 8 1069 1076 10.5588/ijtld.10.0615 21740670

[B18] Hinderaker SG Madland S Ullenes M Enarson DA Rusen ID Kamara D Treatment delay among tuberculosis patients in Tanzania: data from the FIDELIS initiative BMC Public Health 2011 11 306 1 6 21569434 10.1186/1471-2458-11-306 PMC3115859

[B19] Qureshi SA Morkve O Mustafa T Patient and health system delays: health-care seeking behaviour among pulmonary tuberculosis patients in Pakistan J Pak Med Assoc 2008 58 6 318 321 18988391

[B20] Meintjes G Schoeman H Morroni C Wilson D Maartens G Patient and provider delay in tuberculosis suspects from communities with a high HIV prevalence in south Africa: a cross-sectional study BMC Infect Dis 2008 8 72 1 8 18501019 10.1186/1471-2334-8-72 PMC2413241

[B21] Demissie M Lindtjorn B Berhane Y Patient and health service delay in the diagnosis of pulmonary tuberculosis in Ethiopia BioMedCentral Public Health 2002 2 23 1 7 10.1186/1471-2458-2-23 PMC130033 12296975

[B22] Lorent N Mugwaneza P Mugabekazi J Gasana M Van Bastelaere S Risk factors for delay in the diagnosis and treatment of tuberculosis at a referral hospital in Rwanda Int J Tuberc Lung Dis 2008 12 4 392 396 18371264

[B23] Wandwalo ER Morkve O Delay in tuberculosis case-finding and treatment in Mwanza. Tanzania Int J Tuberc Lung Dis 2000 4 2 133 138 10694091

[B24] Mfinanga SG Mutayoba BK Kahwa A Kimaro G Mtandu R Ngadaya E Egwaga S Kitua AY The magnitude and factors associated with delays in management of smear positive tuberculosis in Dar es Salaam. Tanzania BMC Health Serv Res 2008 8 158 10.1186/1472-6963-8-158 18655730 PMC2515835

[B25] Johansson E Long NH Diwan VK Winkvist A Gender and tuberculosis control: perspectives on health seeking behaviour among men and women in Vietnam Health Policy 2000 52 1 33 51 10.1016/S0168-8510(00)00062-2 10899643

[B26] Mills EJ Beyrer C Birungi J Dybul MR Engaging men in prevention and care for HIV/AIDs in Africa Plos Medicine 2012 9 2 e1001167 10.1371/journal.pmed.1001167 22346735 PMC3274499

[B27] Kigozi IM Dobkin LM Martin JN Geng EH Muyindike W Emenyonu NI Bangsberg DR Hahn JA Late-disease stage at presentation to an HIV clinic in the era of free antiretroviral therapy in sub-Saharan Africa J Acquir Immune Defic Syndr 2009 52 2 280 289 10.1097/QAI.0b013e3181ab6eab 19521248 PMC2815238

[B28] WHO Global tuberculosis control: surveillance, planning, financing 2004 Geneva: World Health Organization http://www.who.int/tb/publications/global_report/2004/en/

[B29] WHO TB reach fact sheet 2012 Geneva: Stop TB Partnership Secretariat World Health Organization http://www.stoptb.org/global/awards/tbreach/about.asp

[B30] Boehme CC Nabeta P Hillemann D Nicol MP SS Rapid molecular detection of tuberculosis and rifampin resistance N Engl J Med 2010 363 1005 1015 10.1056/NEJMoa0907847 20825313 PMC2947799

[B31] WHO Tuberculosis diagnostic Xpert MTB/RIF test 2012 Geneva: World Health Organisation http://www.who.int/tb/features_archive/factsheet_xpert.pdf

[B32] Chandra TJ Same day sputum smear microscopy approach for the diagnosis of pulmonary tuberculosis in a microscopy centre at Rajahmundry Indian J Tuberc 2012 59 3 141 144 23362710

